# Saying ‘Thank You’ to those whose thoughts really helped us forward

**DOI:** 10.1007/s40037-017-0368-7

**Published:** 2017-07-24

**Authors:** Jimmie Leppink

**Affiliations:** School of Health Professions Education, Maastricht University, Maastricht, The Netherlands

To the Editor,

This letter is the result of a repeated observation – as an associate/statistical editor, statistical advisor, and author in the field of medical education – that peer reviews are quite often of such a high quality that they help authors resubmit a revised manuscript that, across sections, is a substantially improved version of the original manuscript.

Editorial Boards of journals across fields are fortunate that scholars from a variety of expertise areas spend some of their precious free time reviewing manuscripts. Frequently, they do not just provide a short review with an ‘accept’ or ‘reject’ recommendation but share perspectives, references, and practices that provide authors with great tools to improve their manuscript. The field of medical education is no exception to this.

Luckily, medical education journals include a ‘Thanks to reviewers’ (e. g [1]). , usually in the first issue of a new year, to thank all the people who served as reviewers for the journal in the past year. This is a great thing because it allows us to acknowledge reviewers’ efforts without having to link individual reviewers to specific manuscripts. However, I would like to propose to consider going one step further: to give authors the *explicit *option (e. g. in the Instructions for Authors) of thanking the reviewers in the Acknowledgements section. If a journal’s policy is to keep reviewers anonymous or, in other cases, reviewers have indicated that they do not give consent to their identity being revealed, authors may still want to thank the reviewers anonymously – such as in a recently published manuscript in Health Psychology Review [2] – thereby acknowledging that the peer review process has had a substantial positive impact on the quality of the ultimately accepted manuscript. Moreover, in the cases where reviewers give consent to their identity being revealed, why not give authors the explicit option of mentioning these reviewers in the Acknowledgements?

As an author, I have more often than not seen a manuscript accepted for publication where I realised how much it had been improved just by incorporating the reviewers’ input into the revised version, and this includes some of my own publications in the field of medical education (e. g. [3]). Of course, some might respond that performing reviews is part of a scholar’s job. However, the step that I am proposing is about acknowledging those whose intellectual input has had a profound positive impact on manuscripts that are accepted for publication.
